# Status of the Parkinson’s disease gene family expression in non-small-cell lung cancer

**DOI:** 10.1186/s12957-015-0646-y

**Published:** 2015-08-07

**Authors:** Quan Xing Liu, Hong Zheng, Xu Feng Deng, Dong Zhou, Ji Gang Dai

**Affiliations:** Department of Thoracic Surgery, Xinqiao Hospital, Third Military Medical University, Chongqing, 400037 China; Institute of Immunology of PLA, Third Military Medical University, Chongqing, 400037 China

**Keywords:** Carcinoma, Non-small-cell lung cancer, Parkinson’s disease, Gene, mRNA

## Abstract

**Background:**

The purpose of this study is to detect the Parkinson’s disease gene family mRNA relative expression in the non-small-cell lung cancer (NSCLC) tumor tissue and analyze the association between tumor characteristics and the Parkinson’s disease gene family.

**Methods:**

Tumor tissue and tumor-adjacent tissue of 114 NSCLC patients were collected and SYBR quantitative analysis was used to detect the relative expression level of nine Parkinson’s disease gene mRNAs. Then, paired sample test, two-sided Student’s *t*-test, or two-sided Wilcoxon rank sum test was performed to analyze the mRNA relative expression level of nine Parkinson’s disease gene mRNAs in different gender, tumor histology, and tumor stage.

**Results:**

Overexpression in the tumors was detected in 46/114 (40.35 %) PARK1/4, 74/114 (64.91 %) PARK2, 104/114 (91.23 %) PARK5, 95/114 (83.33 %) PARK6, 80/114 (70.18 %) PARK7, 55/114 (48.25 %) PARK8, 100/114 (87.72 %) PARK9, 55/114 (48.25 %) PARK15, and 99/114 (86.84 %) glucocerebrosidase (GBA). Five genes PARK5 (91.23 %), PARK6 (83.33 %), PARK7 (70.18 %), PARK9 (87.72 %), and GBA (86.84 %) were supposed to be overexpressed in the lung tumor tissues compared with tumor-adjacent tissues. There was no significant difference in PARK1/4, PARK2, PARK5, PARK9, and GBA mRNA expression by different tumor stage, whereas, PARK6, PARK7, PARK8, and PARK15 mRNA expression were found to have significant difference in the comparison of different tumor stages. The expression of PARK6 (*P* = 0.01, *P* = 0.03) and PARK15 (*P* < 0.001, *P* < 0.001) were significantly higher in stages I and II when compared with stage III, respectively. NSCLC patients in stage I showed the higher expression PARK7 compared to the patients in stage II (*P* = 0.003).

**Conclusions:**

The high expression of PARK6, PARK7, and PARK15 might lead to the occurrence of a primary NSCLC tumor, and the tumor with a decreasing expression of these three genes tends to be stages II and III. The results of our study indicate that the Parkinson’s disease gene family may be a potential marker for the prediction of NSCLC.

**Electronic supplementary material:**

The online version of this article (doi:10.1186/s12957-015-0646-y) contains supplementary material, which is available to authorized users.

## Background

Parkinson’s disease is a chronic, progressive neurological disorder caused by the degeneration and death of cells in the substantia nigra [1]. Historically, Parkinson’s disease was regarded as a sporadic disorder, with little or no contribution from hereditary factors. However, over the past 15 years, there has been a step change in our genetic understanding of the condition. SNCA the sodium channel protein para (which encodes α-synuclein) was the first gene to be discovered through linkage analysis in several large kindreds with familial Parkinson’s disease [2]. Recent genome-wide association studies have found that more than a dozen loci (including PARK1/4, PARK2, PARK5, PARK6, PARK7, PARK8, PARK9, PARK15, and glucocerebrosidase (GBA)) have been linked with familial Parkinson’s disease, and currently, upwards of 10 % of all cases of Parkinson’s disease are estimated to be associated with Parkinson’s disease gene family [3–5].

Fifteen years ago, Møller et al. [6] first reported that patients with Parkinson’s disease (PD) seem to have a lower-than-expected rate of lung cancer which has been reaffirmed by many subsequent epidemiological studies [7–10]. This association might account for the overlap function of the Parkinson’s disease gene family between these two apparently unrelated diseases. Recent genetic studies and emerging functional work show that a possible connection between Parkinson’s disease and lung cancer is alluded.

Cancer cells are prone to accumulate mutations, not all of which will necessarily contribute to cancer progression (these are known as passenger mutations, in contrast to the pivotal driver mutations that propel cancerous change). However, when the functional roles of the Parkinson’s disease gene family are considered, a picture emerges of considerable pathogenic overlap between these two apparently unrelated diseases. Currently, several studies have reported that cell systems are affected by the same dysfunctional protein in both Parkinson’s disease and cancer [11–13].

In short, the same mutations can lead either to inappropriate neuronal death in Parkinson’s disease when present in the germ line or to inappropriate cell survival in cancer when present in somatic cells. SNCA (PARK1 or PARK4) was reported overexpressed in brain [14], malignant [15], and ovary tumors [16] while low-expressed or not expressed in those normal and benign tissues. PARK7 (DJ-1) was reported overexpressed in the non-small-cell lung cancer (NSCLC) and prostate cancer cell line [17, 18]. Although many studies have reported that some Parkinson’s disease gene family are overexpressed or low-expressed in brain, malignant, ovary, and colon cancer patients and cell lines, the expression of the Parkinson’s disease gene family in NSCLC patients and the association of clinical data are still unknown.

In this article, we investigated the expression of the Parkinson’s disease gene family in NSCLC patients and further analyzed its association with different tumor histological type, gender, and tumor stage. Our analysis is at times speculative, and additional experimental research may be required to further identify the association between Parkinson’s disease gene family and NSCLC which might help us to discover the new potential oncogenes or tumor suppressors and thus how to treat NSCLC.

## Methods

### Patients

All the 114 NSCLC patients in the study were diagnosed and histopathologically confirmed in our hospital between Feb 2012 and Apr 2013 and without any other cancers or previous chemo- or radiotherapy. The 114 patients were aged 18–65 years old (56.4 ± 10.7) at the time of diagnosis including 50 squamous carcinomas (SQC), and 64 adenocarcinomas (ADC) and were composed of 72 males and 42 females. This study was approved by the institutional review board of Xinqiao Hospital (2012016), and all patients signed informed consent.

### Dispose of tumor tissue and tumor-adjacent tissue

Tumor tissue and tumor-adjacent tissue were collected at the time of surgical resection. All the samples were immediately snap-frozen in liquid nitrogen and stored at −170 °C before use. Total RNA was extracted following a TRIzol extraction protocol (Invitrogen, USA).

### Reverse transcription and SYBR quantitative real-time PCR assay

Total RNA was transcriptioned reversely to cDNA by using PrimeScript™ RT reagent Kit with gDNA Eraser (TAKARA, Japan). SYBR (Synergy BrandsSynergy Brands) quantitative analysis of mRNA expression was used to investigate the expression of Parkinson’s disease gene family, including PARK1/4 (*SCNA*), PARK2 (*Parkin*), PARK5 (*UCHL1*), PARK6 (*PINK1*), PARK7 (*DJ-1*), PARK8 (*LRRK2*), PARK9 (*ATP13A2*), PARK15 (*FBXO7*), and GBA [19], in the tumor tissue and tumor-adjacent tissue. Primers for SYBR q-PCR were designed and synthesized by Sangon Biotech (China) (Table 1). PCR reactions (15 μl) contained 0.25 μl of each primer (10 μM), 7.5 μl SYBR® Premix Ex Taq TM II 2× (TAKARA, Japan), 6 μl ddH_2_O, and 1 μl cDNA. The PCR conditions consisted of an initial denaturation at 95 °C for 30 s followed by amplification for 40 cycles of 15 s at 95 °C and 50 s at 60 °C, with fluorescence acquisition at the end of each extension step. PCR using primers for *β*_*2*_*-microglobulin* was performed on each individual sample as an internal control. The optical density of each PCR band was measured semi-quantitatively using Illumina Eco software (Illumina, San Diego, USA).

**Table 1:** Sequence of primers used in *Parkinson’s disease gene* mRNA real-time q-PCR

Primer	GeneBank ID	Sequence forward (5′–3′)	Sequence reverse (5′–3′)	Product (bp)
PARK1/4 *SCNA*	NM000345.3	AAACCAAGGAGGGAGTGGTG	CTGTCTTCTGGGCTACTGCTG	117
PARK2 *Parkin*	AB009973.1	CTGACACCAGCATCTTCCAG	CCAGTCATTCCTCAGCTCCT	107
PARK5 *UCHL1*	BC000332.2	GCCAATGTCGGGTAGATGAC	AGCGGACTTCTCCTTGCTC	192
PARK6 *PINK1*	AB053323.1	CAAGAGAGGTCCCAAGCAAC	GGCAGCACATCAGGGTAGTC	117
PARK7 *DJ-1*	D61380.2	TGGCTAAAGGAGCAGAGGAA	ATGACCACATCACGGCTACA	127
PARK8 *LRRK2*	AY792511.1	GAGCACGCCTCCAAGTTATT	AGAAGTGACCAACCCACCTG	110
PARK9 *ATP13A2*	AL354615.1	TGGCTGGCTGACCACTACTAC	AGTCTGGCTTTGCTTTCTGG	102
PARK15 *FBXO7*	AF233225.1	TACCCGACAAGCACTGAACC	AAGACGGAACGAACATCCAG	102
*GBA*	D13286.1	CTTCTGCTGGGCTGTTGAGT	TACTGTTGGCGAGGGTAGGA	108
*β2-microglobulin*	NM004048.2	ACCCCCACTGAAAAAGATGA	ATCTTCAAACCTCCATGATG	114

### Statistical analysis

Relative Parkinson’s disease gene family expression was calculated for each patient. The differences in mean value of Parkinson’s disease gene family expression in tumor tissue and tumor-adjacent tissue were analyzed using paired samples test. The differences in gene expression between gender, tumor histology, and tumor stage were analyzed using two-sided Student’s *t*-test or two-sided Wilcoxon rank sum test. All statistical analyses were performed using SPSS 18.0 software (SPSS Inc.), and *P* < 0.05 was considered to be statistically significant.

## Results

### Relative expression level of Parkinson’s disease gene family

**Fig. 1:**
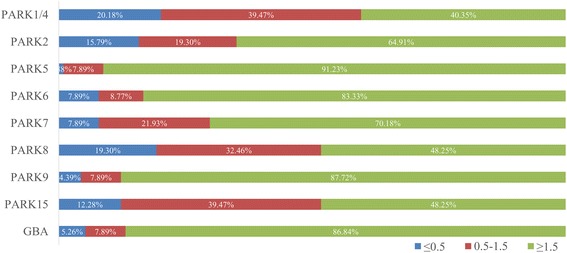
The relative expression of Parkinson’s disease gene mRNA in NSCLC patients. *Blue*, *red*, and *green columns* mean the ≤0.5-fold, 0.5–1.5-fold, and ≥1.5-fold of mRNA relative expression compared with tumor-adjacent tissue, respectively

Expression levels of nine Parkinson’s disease genes and a reference gene were measured in 114 matched pairs of NSCLCs/adjacent histologically normal lung tissue samples by real-time q-PCR. According to the relative mRNA expression fold of nine different genes, patients were divided into five groups (Table. 2). A relative gene expression ratio (T/N) of 1.5 was considered as positive for overexpression in the tumor when compared to the corresponding tumor-adjacent tissue. Values more than 1.5 have been used as the criterion for overexpression of genes in several studies employing proteomic analysis [20]. Therefore, overexpression in the tumors was detected in 46/114 (40.35 %) PARK1/4, 74/114 (64.91 %) PARK2, 104/114 (91.23 %) PARK5, 95/114 (83.33 %) PARK6, 80/114 (70.18 %) PARK7, 55/114 (48.25 %) PARK8, 100/114 (87.72 %) PARK9, 55/114 (48.25 %) PARK15, and 99/114 (86.84 %) GBA. PARK7 (DJ-1) was considered to be overexpressed in primary NSCLC tissues in a previous study [18], and 70.18 % of the patients were detected to have PARK7 overexpression in this study. Then, the five genes PARK5 (91.23 %), PARK6 (83.33 %), PARK7 (70.18 %), PARK9 (87.72 %), and GBA (86.84 %) were supposed to be overexpressed in the lung tumor tissue compared with the tumor-adjacent tissue. Inactivating mutations and deletions of PARK2 have been found in lung cancer [18] and overexpression of PARK15 was also detected in lung squamous cell carcinoma [21]; however, more than half (64.91 %) of the patients showed the overexpression of PARK2, and there was only 48.25 % PARK15-overexpression patients in this study. Their inconsistencies in findings may be caused by the small sample size and different research objects. The relationship between lung cancer and the expression of the other six genes PARK1/4, PARK5, PARK6, PARK8, PARK9, and GBA was first described (Fig. 1).

**Table 2:** The frequency distribution of *Parkinson’s disease gene* mRNA relative expression

Fold	PARK1/4 *SCNA*	PARK2 *Parkin*	PARK5 *UCHL1*	PARK6 *PINK1*	PARK7 *DJ-1*	PARK8 *LRRK2*	PARK9 *ATP13A2*	PARK15 *FBXO7*	*GBA*
	(*n* = 114)	(*n* = 114)	(*n* = 114)	(*n* = 114)	(*n* = 114)	(*n* = 114)	(*n* = 114)	(*n* = 114)	(*n* = 114)
≤0.5	23	18	1	9	9	22	5	14	6
	(20.18 %)	(15.79 %)	(0.88 %)	(7.89 %)	(7.89 %)	(19.30 %)	(4.39 %)	(12.28 %)	(5.26 %)
0.5–1.5	45	22	9	10	25	37	9	45	9
	(39.47 %)	(19.30 %)	(7.89 %)	(8.77 %)	(21.93 %)	(32.46 %)	(7.89 %)	(39.47 %)	(7.89 %)
≥1.5	46	74	104	95	80	55	100	55	99
	(40.35 %)	(64.91 %)	(91.23 %)	(83.33 %)	(70.18 %)	(48.25 %)	(87.72 %)	(48.25 %)	(86.84 %)
≥5	2	19	91	45	14	15	66	9	56
	(1.75 %)	(16.67 %)	(79.82 %)	(39.47 %)	(12.28 %)	(13.16 %)	(57.89 %)	(7.89 %)	(49.12 %)
≥10	0	4	70	16	2	2	39	0	23
	(0.00 %)	(3.51 %)	(61.40 %)	(14.04 %)	(1.75 %)	(1.75 %)	(34.21 %)	(0.00 %)	(20.18 %)

*Fold* means the fold of relative mRNA gene expression to reference gene *β2-microglobulin*

### Associations of tumor characteristics with Parkinson’s disease genes

Mean values of PARK1/4 (*SCNA*), PARK2 (*Parkin*), PARK5 (*UCHL1*), PARK6 (*PINK1*), PARK7 (*DJ-1*), PARK8 (*LRRK2*), PARK9 (*ATP13A2*), PARK15 (*FBXO7*), and GBA relative mRNA expression according to gender, tumor histology, and each clinical stage are summarized in Table 3. Significant statistical difference of relative mRNA expression level of these nine genes was detected in the comparison between tumor tissue and tumor-adjacent tissue. The relative expression of these nine gene mRNAs showed no significant difference between the male groups and female groups. However, the ADC group showed significantly higher PARK2 (*P* < 0.001) and PARK7 (*P* = 0.005) mRNA expression than the SQC group, and no significant difference was found in the other seven genes. There was no significant difference in PARK1/4, PARK2, PARK5, PARK9, and GBA mRNA expression by different tumor stage, whereas PARK6, PARK7, PARK8, and PARK15 mRNA expression were found to have significant difference in the comparison of different tumor stages. The expression of PARK6 (*P* = 0.01, *P* = 0.03) and PARK15 (*P* < 0.001, *P* < 0.001) were significantly higher in stages I and II when compared with stage III, respectively. NSCLC patients in stage I showed the higher expression PARK7 compared to the patients in stage II (*P* = 0.003). Interestingly, the relative expression of PARK8 for the NSCLC patients in stage II was higher than that in stages I (*P* = 0.04) and III (*P* < 0.001).

**Table 3:** Associations of tumor characteristics with *Parkinson’s disease gene* mRNA relative expression in NSCLC

Tumor characteristics	*n* (%)	PARK1/4 (*SCNA*)			PARK2(*Parkin*)			PARK5(*UCHL-1*)			PARK6(*PINK1*)			PARK7(*DJ-1*)			PARK8(*LRRK2*)			PARK9(*ATP13A2*)			PARK15(*FBXO7*)			*GBA*		
		Means ± SD	*P*		Means ± SD	*P*		Means ± SD	*P*		Means ± SD	*P*		Means ± SD	*P*		Means ± SD	*P*		Means ± SD	*P*		Means ± SD	*P*		Means ± SD	*P*	
Overall	114 (100.00%)	1.475 ± 1.088			3.177 ± 3.646			18.375 ± 15.551			5.465 ± 4.949			2.773 ± 2.336			2.272 ± 2.350			8.356 ± 7.402			1.956 ± 1.783			5.936 ± 4.328		
Gender																												
Male	72 (63.16%)	1.499 ± 1.099			3.095 ± 3.974			18.349 ± 16.095			5.566 ± 4.993			2.638 ± 2.079			2.336 ± 2.357			8.329 ± 7.751			2.207 ± 1.938			6.472 ± 4.709		
Female	42 (39.84%)	1.433 ± 1.079	.755^a1^		3.317 ± 3.044	.756^a1^		18.419 ± 14.760	.982^a1^		5.291 ± 4.927	.776^a1^		3.005 ± 2.733	.422^a1^		2.161 ± 2.362	.703^a1^		8.401 ± 6.852	.960^a1^		1.524 ± 1.398	.500^a2^		5.016 ± 3.443	.147^a2^	
Histology																												
Adenocarcinoma	64 (56.14%)	1.584 ± 1.080			4.900 ± 4.200			18.383 ± 14.360			5.422 ± 5.595			3.085 ± 2.710			2.346 ± 2.241			8.904 ± 7.890			2.125 ± 1.945			6.857 ± 4.274		
Squamous cell carcinoma	50 (44.86%)	1.367 ± 1.116	.303^b1^		1.202 ± 1.043	.000^b2^		18.784 ± 17.247	.894^b1^		5.436 ± 3.857	.988^b1^		2.410 ± 1.823	.005^b1^		1.901 ± 1.871	.274^b1^		7.557 ± 6.534	.337^b1^		1.775 ± 1.617	.311^b1^		5.642 ± 3.733	.282^b1^	
Tumor stage																												
Stage I	36 (31.58%)	1.723 ± 1.371	.280^c1^	I–II	5.403 ± 5.442	.040^c2^	I–II	22.808 ± 18.141	.410^c2^	I–II	7.404 ± 6.642	.995^c1^	I–II	4.074 ± 3.377	.003^c2^	I–II	1.631 ± 1.391	.040^c2^	I–II	9.608 ± 9.410	.427^c2^	I–II	3.240 ± 2.546	.070^c2^	I–II	6.440 ± 5.055	.450c1	I–II
Stage II	53 (46.49%)	1.457 ± 0.936	.176^c1^	II–III	2.217 ± 1.783	.598^c1^	II–III	14.625 ± 11.716	3.27^c2^	II–III	5.362 ± 4.101	.010^c2^	II–III	1.894 ± 0.743	.105^c2^	II–III	3.306 ± 2.880	.000^c2^	II–III	6.626 ± 5.645	.180^c1^	II–III	1.567 ± 0.818	.000^c2^	II–III	5.700 ± 4.131	.098c1	II–III
Stage III	25 (21.93%)	1.155 ± 0.854	.720^c1^	I–III	2.001 ± 1.278	.130^c1^	I–III	19.939 ± 17.271	.538^c2^	I–III	2.891 ± 1.406	.003^c2^	I–III	2.765 ± 1.904	.215^c2^	I–III	1.002 ± 1.904	.100^c2^	I–III	10.198 ± 6.880	.790^c1^	I–III	0.930 ± 0.398	.000^c2^	I–III	5.714 ± 3.663	.542c1	I–III

*I–II* means the compare between stages I and II, *II–III* means the compare between stages II and III, *I–III* means the compare between stages I and III

a: Test for equality of medians between different gene relative expression and different sex: ^a1^two-sided Student’s *t*-test; ^a2^two-sided Wilcoxon rank sum test

b: Test for equality of medians between different gene relative expression and different tumor histology: ^b1^two-sided Student’s *t*-test; ^b2^two-sided Wilcoxon rank sum test

c: Test for equality of medians between different gene relative expression and different tumor stage: ^c1^two-sided Student’s *t*-test; ^c2^two-sided Wilcoxon rank sum test

## Discussion

The curious cancer pattern in certain neurological conditions has drawn increasing attention as converging evidence suggests that one family of diseases may provide protection against the other. Over 50 years ago, it was anecdotally noted that patients with Parkinson’s disease (PD) seem to have a lower-than-expected rate for most cancers [22]. Cancer is characterized by unlimited cellular proliferation, while PD is a process of premature cell death. In this sense, the diseases appear to be opposing ends of the same spectrum. In fact, they share many genes and biological pathways, and these are often regulated in different directions. The main cell components (protein degradation, cell cycle, mitochondria, PI3K–AKT–mTOR pathway and inflammation) and the main Parkinson’s-disease-associated proteins (parkin, PTEN-induced putative kinase 1 (PINK1), DJ-1, leucine-rich repeat kinase 2 (LRRK2), glucocerebrosidase (GBA), and F-box protein 7 (FBXO7)) are depicted with the multiple interactions that exist between them.

Neurons and cancer cells are fundamentally different in how they use mitochondria. Whereas neurons use oxidative phosphorylation to generate ATP, cancer cells use glycolysis to a greater extent, which is partly explained by the hypoxic tumor environment. However, this alternative metabolism persists in tumor cells even in the presence of oxygen, as originally observed by Warburg nearly a century ago [23]. So, mitochondrial dysfunction has long been implicated in the development of cancer, and this perspective is now undergoing a renaissance. Functional studies of Parkinson’s disease genes indicate that the mTOR (mammalian target of rapamycin) pathway probably has a major bearing on neurodegeneration [24–26]. The mTOR pathway is a central regulator of cell growth and proliferation that mainly functions through the modulation of protein synthesis. The central importance of this pathway for cancer biology is reflected by the fact that an mTOR inhibitor, sirolimus (also known as rapamycin), is already in use in oncology practice, and several trials of PI3K and AKT inhibitors are underway [27–29]. Inflammation promotes tumorigenesis and progression by providing growth factors that sustain proliferative signalling and survival factors that limit cell death. Chronic inflammation is regarded as an enabling characteristic in cancer, and recent work suggests that a similar mechanism might drive pathological change in PD conditions [30, 31].

To date, only one study has examined the expression of the PARK7 in primary NSCLCs [18]. However, no comparison was made between expression levels in matched tumor/normal pairs for the Parkinson’s disease gene family. The objective of the current study was to determine whether the Parkinson’s disease gene family is overexpressed in NSCLCs as compared to adjacent histologically normal lung tissue samples and further determine the relationship of the expression levels to gender, tumor sub-type, and tumor stage. One hundred and fourteen matched human NSCLC tumor/normal pairs were examined by real-time q-PCR analysis for expression of PARK1/4 (SCNA), PARK2 (Parkin), PARK5 (UCHL1), PARK6 (PINK1), PARK7 (DJ-1), PARK8 (LRRK2), PARK9 (ATP13A2), PARK15 (FBXO7), and GBA mRNA. Using a value of 1.5 as the criterion for mRNA overexpression, elevated levels of Parkinson’s disease gene family mRNA in the NSCLC were detected and performed in Table 2. PARK7 (DJ-1) was considered to be overexpressed in primary NSCLC tissues in a previous study, and 70.18 % of the patients were detected to have PARK7 overexpression in this study. Then, the five genes PARK5 (91.23 %), PARK6 (83.33 %), PARK7 (70.18 %), PARK9 (87.72 %), and GBA (86.84 %) were supposed to be overexpressed in the lung tumor tissue compared with the tumor-adjacent tissue. There was no apparent correlation between the Parkinson’s disease gene family ratio (T/N) of different genders. PARK6, PARK7, PARK8, and PARK15 mRNA expression were found to have significant difference in the comparison of different tumor stages. The high expression of PARK6, PARK7, and PARK15 might lead to the occurrence of a primary tumor, but the tumor with a decreasing expression of PARK6 and PARK15 tends to be the stages II and III tumor. Only PARK2 and PARK7 in the ADC group showed significantly higher mRNA expression than those in the SQC group, which revealed that PARK2 and PARK7 may play a more important role in normal lung cells that transform into adenocarcinomas than those that become squamous cell carcinomas. Adjacent normal lung tissue is the best control we could obtain for this study. Although the tissue was observed to be histologically normal, it is unclear what molecular changes have occurred in these cells and, therefore, how far along these cells are with respect to developing into cancer cells.

## Conclusions

The data presented in this manuscript suggest that further characterization of the cells that comprise both lung tumors and adjacent histologically normal tissue is warranted to establish the molecular mechanisms resulting in overexpression of the Parkinson’s disease gene family. Overall, the results of our study indicate that the Parkinson’s disease gene family may be a potential marker for the prediction of NSCLC. In a reciprocal manner, functional studies in a Parkinson’s disease context may provide novel insights into the role of cancer-associated genes. Ultimately, perhaps there will be therapeutic agents that can target both conditions.

## Additional file

Additional file 1:
**Results of** ***t*** **-test for gender and histology.**
