# Supplementary material for: Status of the Parkinson’s disease gene family expression in non-small-cell lung cancer
Source: World J Surg Oncol. 2015 Aug 7;13:238. doi: 10.1186/s12957-015-0646-y (PMC4527104; doi:10.1186/s12957-015-0646-y)
Supplement: Additional file 1: — Results of t -test for gender and histology. [file 12957_2015_646_MOESM1_ESM.docx]

Results of t-test for gender

PARK1/4 SCNA

| **组统计量** | | | | | |  |  |  |  |  |
| --- | --- | --- | --- | --- | --- | --- | --- | --- | --- | --- |
|  | Gender | N | mean | 标准差 | 均值的标准误 |  |  |  |  |  |
| 数值 | male | 72 | 1.4993 | 1.09942 | .12957 |  |  |  |  |  |
|  | female | 42 | 1.4331 | 1.07880 | .16646 |  |  |  |  |  |
|  |  |  |  |  |  |  |  |  |  |  |
| **独立样本检验** | | | | | | | | | | |
|  | | 方差方程的 Levene 检验 | | 均值方程的 t 检验 | | | | | | |
|  |  | F | Sig. | t | df | Sig.( two-tailed) | 均值差值 | 标准误差值 | 差分的 95% 置信区间 | |
|  |  |  |  |  |  |  |  |  | 下限 | 上限 |
| 数值 | 假设方差相等 | .587 | **.445** | .312 | 112 | **.755** | .06621 | .21201 | -.35385 | .48628 |
|  | 假设方差不相等 |  |  | .314 | 87.237 | .754 | .06621 | .21094 | -.35305 | .48547 |

PARK2 Parkin

| **组统计量** | | | | | |  |  |  |  |  |
| --- | --- | --- | --- | --- | --- | --- | --- | --- | --- | --- |
|  | Gender | N | mean | 标准差 | 均值的标准误 |  |  |  |  |  |
| 数值 | male | 72 | 3.0957 | 3.97396 | .46834 |  |  |  |  |  |
|  | female | 42 | 3.3167 | 3.04380 | .46967 |  |  |  |  |  |
|  |  |  |  |  |  |  |  |  |  |  |
| **独立样本检验** | | | | | | | | | | |
|  | | 方差方程的 Levene 检验 | | 均值方程的 t 检验 | | | | | | |
|  |  | F | Sig. | t | df | Sig.( two-tailed) | 均值差值 | 标准误差值 | 差分的 95% 置信区间 | |
|  |  |  |  |  |  |  |  |  | 下限 | 上限 |
| 数值 | 假设方差相等 | .479 | **.490** | -.311 | 112 | **.756** | -.22097 | .71082 | -1.62937 | 1.18743 |
|  | 假设方差不相等 |  |  | -.333 | 103.805 | .740 | -.22097 | .66327 | -1.53629 | 1.09435 |

PARK6 PINK1

| **组统计量** | | | | | |  |  |  |  |  |
| --- | --- | --- | --- | --- | --- | --- | --- | --- | --- | --- |
|  | Gender | N | mean | 标准差 | 均值的标准误 |  |  |  |  |  |
| 数值 | male | 72 | 5.5663 | 4.99342 | .58848 |  |  |  |  |  |
|  | female | 42 | 5.2907 | 4.92683 | .76023 |  |  |  |  |  |
|  |  |  |  |  |  |  |  |  |  |  |
| **独立样本检验** | | | | | | | | | | |
|  | | 方差方程的 Levene 检验 | | 均值方程的 t 检验 | | | | | | |
|  |  | F | Sig. | t | df | Sig.( two-tailed) | 均值差值 | 标准误差值 | 差分的 95% 置信区间 | |
|  |  |  |  |  |  |  |  |  | 下限 | 上限 |
| 数值 | 假设方差相等 | .103 | **.748** | .286 | 112 | **.776** | .27554 | .96481 | -1.63612 | 2.18719 |
|  | 假设方差不相等 |  |  | .287 | 86.849 | .775 | .27554 | .96138 | -1.63536 | 2.18643 |

PARK8 LRRK2

| **组统计量** | | | | | |  |  |  |  |  |
| --- | --- | --- | --- | --- | --- | --- | --- | --- | --- | --- |
|  | Gender | N | mean | 标准差 | 均值的标准误 |  |  |  |  |  |
| 数值 | male | 72 | 2.3361 | 2.35691 | .27776 |  |  |  |  |  |
|  | female | 42 | 2.1610 | 2.36160 | .36440 |  |  |  |  |  |
|  |  |  |  |  |  |  |  |  |  |  |
| **独立样本检验** | | | | | | | | | | |
|  | | 方差方程的 Levene 检验 | | 均值方程的 t 检验 | | | | | | |
|  |  | F | Sig. | t | df | Sig.( two-tailed) | 均值差值 | 标准误差值 | 差分的 95% 置信区间 | |
|  |  |  |  |  |  |  |  |  | 下限 | 上限 |
| 数值 | 假设方差相等 | .012 | **.914** | .382 | 112 | **.703** | .17516 | .45795 | -.73222 | 1.08254 |
|  | 假设方差不相等 |  |  | .382 | 85.765 | .703 | .17516 | .45820 | -.73574 | 1.08606 |

Results of t-test for Histology

PARK1/4 SCNA

| **组统计量** | | | | | |  |  |  |  |  |
| --- | --- | --- | --- | --- | --- | --- | --- | --- | --- | --- |
|  | Histology | N | mean | 标准差 | 均值的标准误 |  |  |  |  |  |
| 数值 | SCC | 50 | 1.3674 | 1.11571 | .15778 |  |  |  |  |  |
|  | ADC | 64 | 1.5838 | 1.08003 | .13828 |  |  |  |  |  |
|  |  |  |  |  |  |  |  |  |  |  |
| **独立样本检验** | | | | | | | | | | |
|  | | 方差方程的 Levene 检验 | | 均值方程的 t 检验 | | | | | | |
|  |  | F | Sig. | t | df | Sig.( two-tailed) | 均值差值 | 标准误差值 | 差分的 95% 置信区间 | |
|  |  |  |  |  |  |  |  |  | 下限 | 上限 |
| 数值 | 假设方差相等 | .211 | **.647** | -1.035 | 109 | **.303** | -.21637 | .20912 | -.63085 | .19811 |
|  | 假设方差不相等 |  |  | -1.031 | 103.375 | .305 | -.21637 | .20981 | -.63245 | .19971 |

PARK6 PINK1

| **组统计量** | | | | | |  |  |  |  |  |
| --- | --- | --- | --- | --- | --- | --- | --- | --- | --- | --- |
|  | Histology | N | mean | 标准差 | 均值的标准误 |  |  |  |  |  |
| 数值 | SCC | 50 | 5.4360 | 3.85710 | .54548 |  |  |  |  |  |
|  | ADC | 64 | 5.4220 | 5.59518 | .71639 |  |  |  |  |  |
|  |  |  |  |  |  |  |  |  |  |  |
| **独立样本检验** | | | | | | | | | | |
|  | | 方差方程的 Levene 检验 | | 均值方程的 t 检验 | | | | | | |
|  |  | F | Sig. | t | df | Sig.( two-tailed) | 均值差值 | 标准误差值 | 差分的 95% 置信区间 | |
|  |  |  |  |  |  |  |  |  | 下限 | 上限 |
| 数值 | 假设方差相等 | .462 | **.498** | .015 | 109 | **.988** | .01403 | .93303 | -1.83521 | 1.86328 |
|  | 假设方差不相等 |  |  | .016 | 106.079 | .988 | .01403 | .90042 | -1.77112 | 1.79919 |

PARK8 LRRK2

| **组统计量** | | | | | |  |  |  |  |  |
| --- | --- | --- | --- | --- | --- | --- | --- | --- | --- | --- |
|  | Histology | N | 均值 | 标准差 | 均值的标准误 |  |  |  |  |  |
| 数值 | SCC | 50 | 1.9012 | 1.87084 | .26458 |  |  |  |  |  |
|  | ADC | 64 | 2.3456 | 2.24052 | .28687 |  |  |  |  |  |
|  |  |  |  |  |  |  |  |  |  |  |
| **独立样本检验** | | | | | | | | | | |
|  | | 方差方程的 Levene 检验 | | 均值方程的 t 检验 | | | | | | |
|  |  | F | Sig. | t | df | Sig.( two-tailed) | 均值差值 | 标准误差值 | 差分的 95% 置信区间 | |
|  |  |  |  |  |  |  |  |  | 下限 | 上限 |
| 数值 | 假设方差相等 | .663 | **.417** | -1.098 | 109 | **.274** | -.43637 | .39727 | -1.22376 | .35101 |
|  | 假设方差不相等 |  |  | -1.118 | 108.955 | .266 | -.43637 | .39025 | -1.20984 | .33709 |
